# Experimental mitochondria-targeted DNA methylation identifies GpC methylation, not CpG methylation, as potential regulator of mitochondrial gene expression

**DOI:** 10.1038/s41598-017-00263-z

**Published:** 2017-03-14

**Authors:** Monique G. P. van der Wijst, Amanda Y. van Tilburg, Marcel H. J. Ruiters, Marianne G. Rots

**Affiliations:** Department of Pathology and Medical Biology, University of Groningen, University Medical Center Groningen (UMCG), Hanzeplein 1, 9713 GZ Groningen, The Netherlands

## Abstract

Like the nucleus, mitochondria contain their own DNA and recent reports provide accumulating evidence that also the mitochondrial DNA (mtDNA) is subjective to DNA methylation. This evidence includes the demonstration of mitochondria-localised DNA methyltransferases and demethylases, and the detection of mtDNA methylation as well as hydroxymethylation. Importantly, differential mtDNA methylation has been linked to aging and diseases, including cancer and diabetes. However, functionality of mtDNA methylation has not been demonstrated. Therefore, we targeted DNA methylating enzymes (modifying cytosine in the CpG or GpC context) to the mtDNA. Unexpectedly, mtDNA gene expression remained unchanged upon induction of CpG mtDNA methylation, whereas induction of C-methylation in the GpC context decreased mtDNA gene expression. Intriguingly, in the latter case, the three mtDNA promoters were differentially affected in each cell line, while cellular function seemed undisturbed. In conclusion, this is the first study which directly addresses the potential functionality of mtDNA methylation. Giving the important role of mitochondria in health and disease, unravelling the impact of mtDNA methylation adds to our understanding of the role of mitochondria in physiological and pathophysiological processes.

## Introduction

For many decades already, the existence of mitochondrial DNA (mtDNA) methylation has been the subject of debate^1–8^. Especially in the early days, the, on average, low level of mtDNA methylation (2–5%)^3, 9^ may have complicated its detection. Moreover, nuclear contamination of isolated mitochondria and the subsequent detection of nuclear integrations of mtDNA (NUMTs) may have distorted the readout. Some recent papers indeed reject the existence of mtDNA methylation^6, 7^. Intriguingly, at the same time, emerging evidence based on a wide variety of techniques^10^, convincingly supports the existence of mtDNA methylation. Such supporting evidence, as reviewed by us elsewhere^11^, includes the discovery of a) a mitochondria-targeted human DNA methyltransferase 1 transcript variant (mtDNMT1)^12^, b) the presence of both CpG and CpH (where H is A, T or C) methylation^8, 12–15^ and, importantly, c) correlations with diseases such as cancer^16^, Down syndrome^17^ and diabetes^18^. Although several of these papers hint toward an effect of mtDNA methylation on mitochondrial gene expression^12, 16, 18–20^, a direct causal link has yet to be demonstrated.

**Figure 1:**
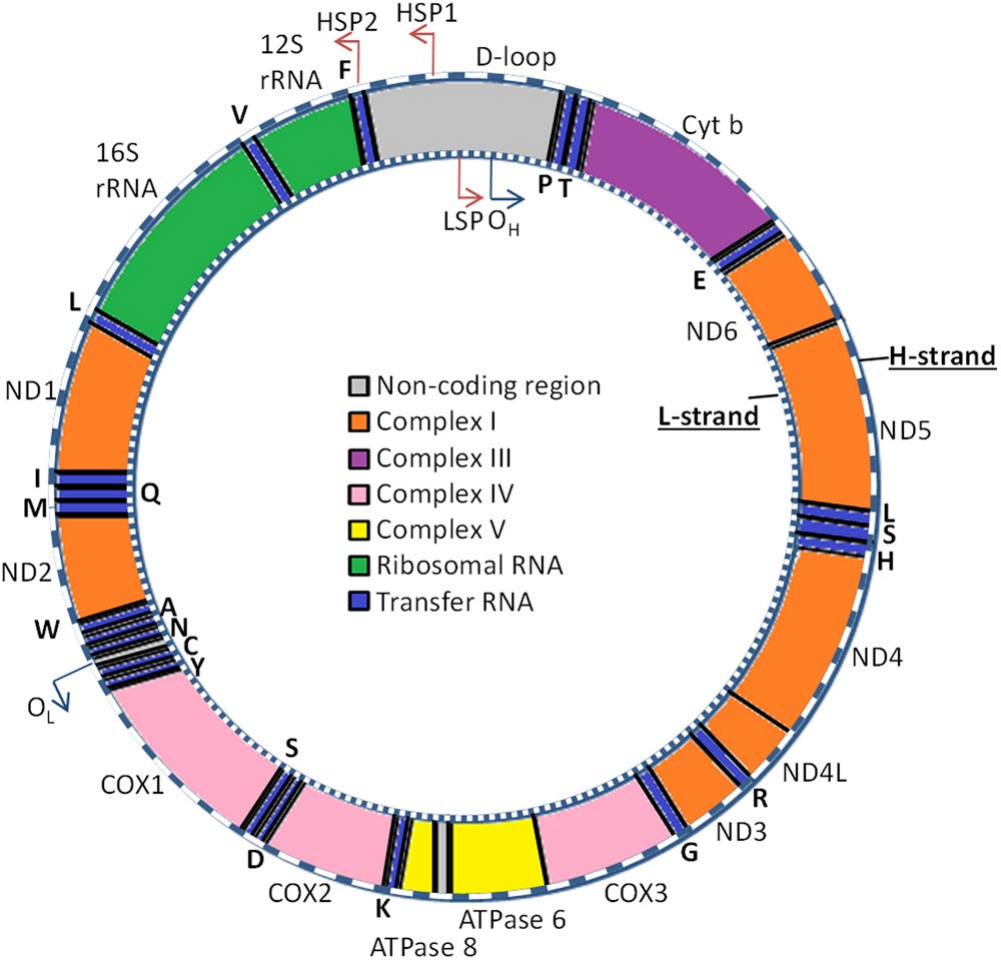
Mitochondrial DNA (mtDNA). The human mtDNA is a 16,569 bp circular DNA, containing a heavy (H, outer ring) and light (L, inner ring) strand. The genes encoded from the L-strand are written inside the circular DNA, whereas genes encoded from the H-strand are written on the outside. The protein-coding genes encode for the complexes required for oxidative phosphorylation (Complex I: orange, complex III: purple, complex IV: pink, complex V: yellow). The D-loop region contains the promoters for the L- and H-strand (LSP, HSP1, HSP2) and the origin of replication of the H-strand (O_H_).

Mitochondrial transcription is differently regulated compared to its nuclear counterpart^21^, and therefore, the effect of mtDNA methylation may be different from the effects known for nuclear DNA (nDNA) methylation. The mtDNA contains one non-coding region called the D-loop control region. It is within or near this region that all three promoters are located: one for the light (L)-strand (LSP), and two for the heavy (H)-strand (HSP1 and HSP2). The LSP and HSP2 give rise to one polycistronic transcript from the L- or H-strand, respectively. The HSP1 gives rise to a short transcript containing rRNA genes (12S and 16S rRNA), whereas LSP and HSP2 encode together for 13 protein-coding genes involved in the oxidative phosphorylation (OXPHOS) and 22 transfer RNAs (tRNAs) (Fig. 1)^22^. Resulting from the above, an effect on mitochondrial gene expression is expected to translate to dysfunctional OXPHOS.

MtDNA methylation may directly regulate mtDNA gene expression (as described above), or alternatively, some suggested that it may do so indirectly^23, 24^ via the modulation of mtDNA replication^13, 15^. MtDNA replication begins with the transcription of a small (~100 bp) RNA strand (7S RNA) from the LSP. This 7S RNA molecule is terminated in the conserved sequence boxes 1–3 and remains bound to the L-strand from which it is synthesised^25^. This event may initiate the transcription of small stretches of the complementary H-strand around the origin of H-strand replication (O_H_) by the mitochondrial DNA polymerase (POLG), resulting in the formation of a short DNA fragment (7S DNA) that together with the mtDNA forms a stable D-loop structure^26, 27^. Interestingly, it is in this region of the D-loop that Bianchessi *et al.* observed the highest methylation frequency and greatest asymmetry of CpG and CpH methylation between both strands^15^. These findings point to a possible functional effect of mtDNA methylation on 7S DNA and/or D-loop formation. The D-loop provides an open DNA structure^28, 29^, which may increase the binding of proteins involved in mtDNA replication or transcription. Therefore, by affecting the accessibility of the D-loop, D-loop mtDNA methylation may indirectly affect these processes.

Despite recent progress in the field of mtDNA methylation and its possible contribution to disease, clear-cut evidence for its functionality is still lacking. Therefore, this study aims to gain insight into functional effects of mtDNA methylation, if present at all. We hypothesize that differential mtDNA cytosine methylation affects mtDNA gene expression or mtDNA replication, and as such may contribute to the pathogenesis of various diseases^14, 16–19^. Here, we show that low levels of methylation can be detected in the mtDNA of various cancer cell lines and fibroblasts of a mtDNA disease patient. Moreover, we provide the first insights into the possible role of both CpG and GpC mtDNA methylation using a mitochondria-targeted bacterial CpG methyltransferase M.SssI (MLS-M.SssI)^30^ and Chlorella virus NYs-1 GpC methyltransferase M.CviPI (MLS-M.CviPI) which methylates Cs in the GpC-context, independent of the 3′ neighbouring nucleotide, so including, but not limited to, CpGs^31^.

## Results

### Detection of mitochondrial DNA methylation

**Figure 2:**
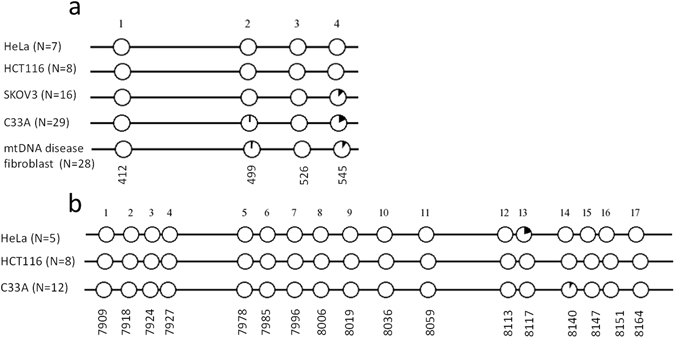
Bisulfite sequencing of mtDNA. Bisulfite sequencing of a region in the D-loop (H-strand) (**a**) and *mtCOX2* gene (L-strand) (**b**) for SKOV3, skin fibroblasts of a mtDNA disease patient (only in **a**) HeLa, HCT116 and C33A cells (**a**,**b**). Each circle represents a single CpG position, the percentage of black of each circle represents the percentage of methylation.

First, the presence and level of mtDNA methylation were determined in two regions that were previously described to be methylated^12, 13^. For this purpose, bisulfite sequencing was performed of a region in the D-loop (Fig. 2a) and *mtCOX2* gene (Fig. 2b) in three to four different cancer cell lines (HeLa, HCT116, SKOV3 and C33A). As shown in Fig. 2, no mtDNA methylation was detected for the majority of analysed CpGs. Nevertheless, 2 out of 4 CpGs in the D-loop region, and 2 out of 17 CpGs in the *mtCOX2* gene did show DNA methylation, albeit at low levels. In the D-loop region, CpG #2 and/or CpG #4 were found to be methylated in C33A and SKOV3, respectively, up to about 3% (1/29 clones) and 17% (5/29 clones) (Fig. 2a). A comparable methylation pattern (4% (1/28 clones) methylation at CpG #2, 11% (3/28 clones) methylation at CpG #4) was found in skin fibroblasts isolated from a patient with a mitochondrial disease (Fig. 2a). In the *mtCOX2* gene, methylation was detected up to 20% (1/5 clones) for CpG #13 and 8% (1/12 clones) for CpG #14 (Fig. 2b). Since several studies show the presence of CpH methylation in the mtDNA^13–15^, also the level of CpH methylation was analysed for our cell lines in both regions. This analysis revealed an average level of CpH methylation below 1% (Suppl. Table 1).

### Mitochondria-targeted DNA methyltransferases efficiently methylate the mtDNA

Next, we addressed the function of mtDNA methylation by inducing methylation via two different approaches. The first approach was to reproduce a published disease model (diabetic retinopathy) in which glucose-induced mtDNA methylation was observed^18^. The second approach was to enforce mtDNA methylation by targeting various DNA methyltransferases to the mitochondria.

For the first approach, we aimed to reproduce the study of Mishra *et al.*, in which a 4 day high (20 mM) versus low (5 mM) glucose treatment in bovine retinal endothelial cells was sufficient to induce DNMT1-mediated mtDNA methylation in the D-loop (3 fold) and *mtCYTB* region (1.8 fold), but not in the *mtCOX2* region^18^. For this purpose, we exposed a variety of healthy (CiGenCs, IHH, OSE-C2) and cancerous (C33A, HCT116) cell lines to high (25 mM) versus low (5 mM) glucose medium for 4 days. Subsequently, methylation of the mtDNA in the D-loop, *mtCOX2* and *mtCYTB* region was determined by MeDIP. None of the analysed regions showed a differential methylation level upon high versus low glucose treatment (Suppl. Fig. 1). Therefore, we continued with our second approach.

For our second approach, we stably expressed mitochondria-targeted DNA methyltransferases, modifying cytosine in the CpG context (MLS-M.SssI: bacterial CpG methyltransferase^30^, MLS-DNMT1: human CpG methyltransferase) or in a different context (MLS-M.CviPI: Nys-1 chorella virus GpC_me_ methyltransferase^31^, MLS-hM.CviPII: humanised Nys-1 chorella virus C_me_CD methyltransferase^32^), in C33A and HCT116 cells. Despite using previously published^12^ (and unpublished) primers (Table 1), we could not clone or detect mRNA expression of the endogenous mitochondria-targeted DNMT1 variant (mtDNMT1), which precluded its direct use. As an alternative, we targeted the normal (non-mitochondria-targeted) *DNMT1* gene to the mitochondria using our mitochondria-targeting plasmid (MLS-DNMT1). Moreover, since it is unknown which methyltransferase may perform CpH methylation of the mtDNA (DNMT1, DNMT3A and DNMT3B have been excluded^13^), mitochondria-targeted viral methyltransferases (MLS-M.CviPI and MLS-hM.CviPII) were used. As a negative control, wild-type cells or cells stably expressing the targeting plasmid without effector domain were generated (MLS-NoED). These cell lines were chosen because of their differential p53 status (C33A – p53 mutant, HCT116 – p53 wild-type), as p53 knockdown is known to preferentially activate mtDNMT1^12^. Moreover, the HCT116 cells were previously used to show that preferential upregulation of mtDNMT1 (by knockdown of p53) could induce expression of mtND1 and repress expression of mtND6^12^.

**Table 1:** Primer sequences.

Target	Forward sequence (5′–3′)	Reverse sequence (5′–3′)
**Cloning**		
AscI-DNMT1-PacI	ataGGCGCGCCATGCCGGCGCGTACCG	cagTTAATTAAGTCCTTAGCAGCTTCCTCCTCCTT
BclI-mtDNMT1-NotI	gtaTGATCACCATGGCCGGCTCCGT	ctaGCGGCCGCCTAGTCCTTAGCAGCTTCCTC
AscI-M.CviPI-PacI	aatGGCGCGCCACCTTGAAAGCGCTCG	ggcTTAATTAATATTCTAACAAATTTCCTAAATATTCTTTG
AscI-hM.CviPII-PacI	taaGGCGCGCCATGAGAACCAAGTATCGGATC	tggTTAATTAAGTAGTGCATCAGGTCCC
NotI-conII promoter-NotI	GGCCGCAGATCCATTATACGAGCCGATGATTAATTGTCAACAGC	GGCCGCTGTTGACAATTAATCATCGGCTCGTATAATGGATCTGC
**q(RT-)PCR**		
mtND1 (RtprimerDB)	ATACCCCCGATTCCGCTACGAC	GTTTGAGGGGGAATGCTGGAGA
mtND6^51^	GGGTGGTGGTTGTGGTAAAC	CCCCGAGCAATCTCAATTAC
mtCOX1 (RtprimerDB)	CGATGCATACACCACATGAA	AGCGAAGGCTTCTCAAATCA
mtCYTB (RtprimerDB)	AATTCTCCGATCCGTCCCTA	GGAGGATGGGGATTATTGCT
12S rRNA^52^	CTGCTCGCCAGAACACTACG	TGAGCAAGAGGTGGTGAGGT
16S rRNA^52^	GTATGAATGGCTCCACGAGG	GGTCTTCTCGTCTTGCTGTG
PGC1α	TGAGAGGGCCAAGCAAAG	ATAAATCACACGGCGCTCTT
NRF1^23^	GGGAGCTACAGTCACTATGG	TCCAGTAAGTGCTCCGAC
TFAM^23^	CCGAGGTGGTTTTCATCTGT	TCCGCCCTATAAGCATCTTG
mtDNMT1 #1^12^	TCCCTGGGCATGGCCGGCT	CTCTTTCCAAATCTTGAGCCGC
mtDNMT1 #2	CCTCCCCATCGGTTTCCG	CCAAATCTTTGAGCCGCCTG
mtDNMT1 #3	ATGGCCGGCTCCGTTCCA	“ ”
β-actin	CCAACCGCGAGAAGATGA	CCAGAGGCGTACAGGGATAG
mtDNA ratio D-loop	TCACCCTATTAACCACTCACGG	ATACTGCGACATAGGGTGCTC
nDNA ratio β-actin	TGAGTGGCCCGCTACCTCTT	CGGCAGAAGAGAGAACCAGTGA
7S DNA primer A + B1^15^	GTGGCTTTGGAGTTGCAGTT	CAGCCACCATGAATATTGTAC
A + B2^15^	“ ”	GAAGCAGATTTGGGTACCAC
**MeDIP**		
D-loop_qMeDIP^18^	ACATAGGGTGCTCCGGCTCCA	TCCGACATCTGGTTCCTACTTCAGG
mtCYTB_qMeDIP^18^	TCACCAGACGCCTCAACCGC	GCCTCGCCCGATGTGTAGGA
mtCOX2_qMeDIP^18^	CCGTCTGAACTATCCTGCCC	GAGGGATCGTTGACCTCGTC
GAPDH_qMeDIP^60^	CTCTCTCCCATCCCTTCTCC	CAAGTTGCCTGTCCTTCCTA
**Bisulfite sequencing**		
BS6_D-loop (H)^13^	CACATCTCTACCAAACCCC	TGGGGTGATGTGAGTTTGTT
BS6_D-loop (L)^13^	**AGAGAG**TATATTTTTGTTAAATTTT	**AGGAAGAGAG**ACCCATCTAAACATTTTCAA
mtCOX2 (L)^7^	ATTGGTTATTAATGGTATTGAATTTA	CTCCACAAATTTCAAAACATTAAC

Restriction sites are underlined.

The 6- to 10-bp tags added to primers are indicated in bold.

**Figure 3:**
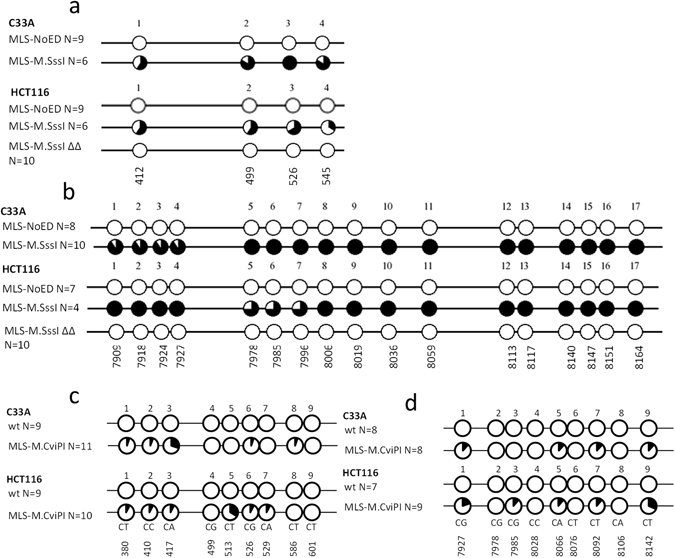
Bisulfite sequencing of mtDNA in cells with or without mitochondria-targeted M.SssI or M.CviPI. Bisulfite sequencing of a region in the D-loop (H-strand) (**a**,**c**) and *mtCOX2* gene (L-strand) (**b**,**d**) for C33A and HCT116 cells expressing a mitochondria-targeted CpG methyltransferase M.SssI (MLS-M.SssI), the catalytically inactive double mutant of M.SssI (MLS-M.SssI ∆∆, only for HCT116 cells) or empty vector control (MLS-NoED) (**a**,**b**), or a mitochondria-targeted GpC methyltransferase M.CviPI or wild-type cells (wt) (**c**,**d**). Each circle represents a CpG (**a**,**b**) or GpC (**c**,**d**) position. The percentage of methylation on each position is represented in black.

To confirm efficient methylation of the mtDNA, two regions (D-loop, *mtCOX2*) in the mtDNA were selected for bisulfite sequencing. We could not detect induction of mtDNA methylation using MLS-DNMT1 or hM.CviPII (data not shown). On the other hand, M.SssI and M.CviPI could both successfully induce mtDNA methylation (Fig. 3). In the D-loop region, M.SssI induced CpG methylation ranging between 60–100% and 33–67% for C33A and HCT116 cells, respectively (Fig. 3a). In the *mtCOX2* region, induction of CpG methylation levels varied between 91–100% and 75–100% for C33A and HCT116 cells, respectively (Fig. 3b). These data were independently confirmed in the HCT116 cells for three mtDNA regions (D-loop, *mtCOX2*, *mtCYTB*) using a methylated DNA immunoprecipation (MeDIP) approach. In line with the bisulfite sequencing data, the MeDIP showed more efficient methylation of the *mtCOX2* gene (~79× induction over IgG) compared to the D-loop (~26× induction over IgG) (Suppl. Fig. 1). Moreover, a catalytically inactive double mutant of M.SssI (MLS-M.SssI ∆∆) was unable to methylate the D-loop (Fig. 3a) or *mtCOX2* region (Fig. 3b) of HCT116 cells. This clearly shows that the observed methylation was dependent on the DNA methyltransferase activity of M.SssI. M.CviPI also successfully methylated the mtDNA in both the D-loop (Fig. 3c) and *mtCOX2* region (Fig. 3d), albeit with lower efficiency than M.SssI. In the D-loop, M.CviPI induced GpC methylation varying between 0–36% and 0–40% for C33A and HCT116 cells, respectively (Fig. 3c). In the *mtCOX2* region, induction of GpC methylation levels ranged between 0–13% and 0–33% for C33A and HCT116 cells, respectively (Fig. 3d).

**Figure 4:**
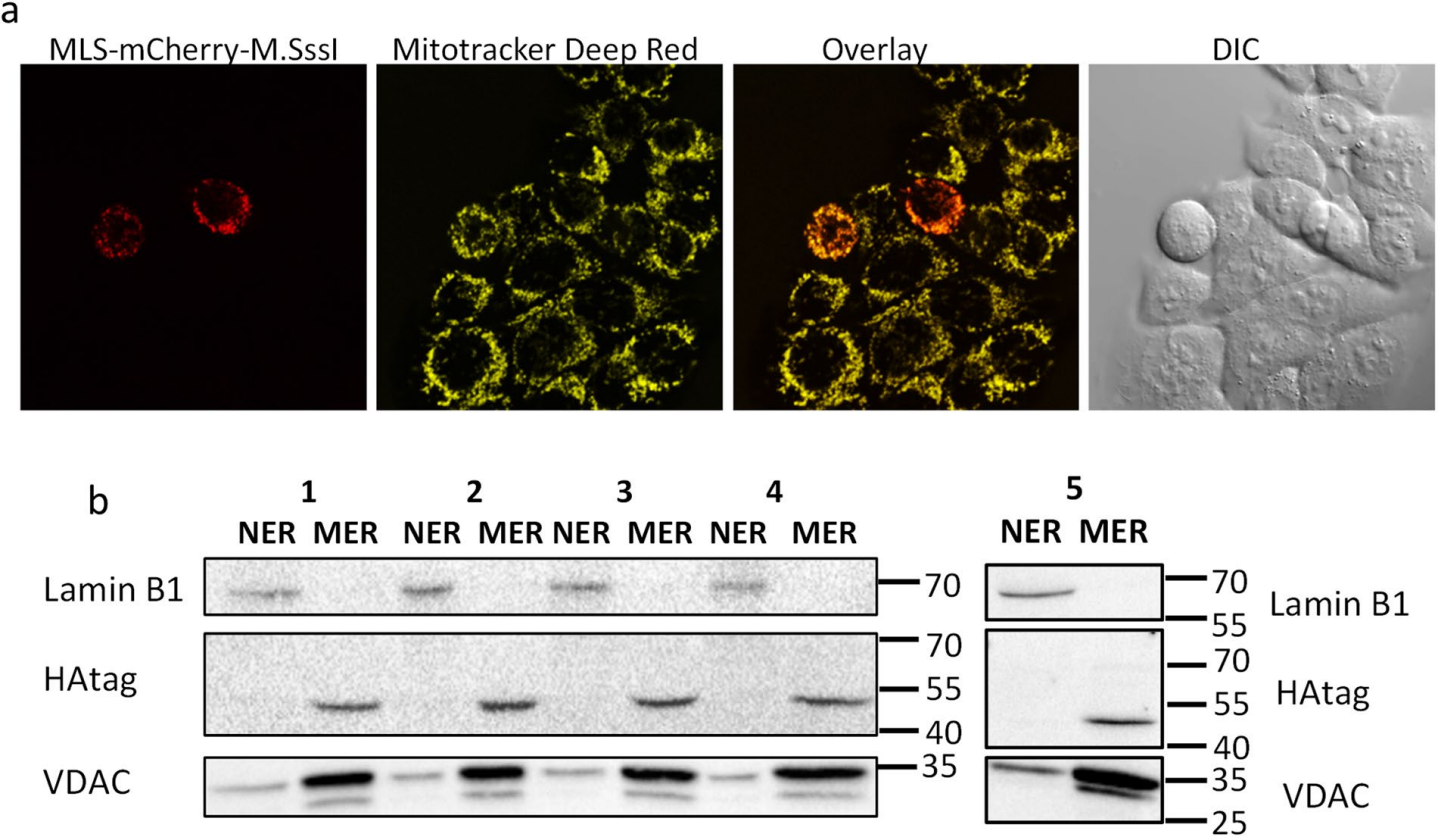
Mitochondrial localization of mitochondria-targeted DNA methyltransferases. (**a**) Confocal microscopy of HCT116 cells expressing MLS-mCherry-M.SssI. In order to stain the mitochondria, cells were incubated at 37 °C for 30 min. with 100 nM Mitotracker Deep Red. (**b**) Western blot of mitochondria-targeted M.CviPI, M.SssI or the catalytically inactive M.SssI ∆∆. Mitochondrial (MER) and nuclear (NER) protein extracts were isolated from C33A cells expressing mitochondria-targeted M.CviPI (lane 1) or M.SssI (lane 5) and HCT116 cells expressing mitochondria-targeted M.CviPI (lane 2), M.SssI (lane 3) or M.SssI ∆∆ (lane 4). A HAtag antibody was used to recognize the mitochondria-targeted constructs in the MER (49 kDa for M.CviPI, 52 kDa for M.SssI) or NER. Inside the mitochondria the mitochondrial-localization signal is cleaved off, reducing the size of the protein with 5 kDa. VDAC1/Porin (32 kDa) and Lamin B1 (68 kDa) were used as mitochondria and nuclear loading controls, respectively.

Exclusive mitochondrial localization of our mitochondria-targeted plasmid was confirmed by confocal microscopy (HCT116 MLS-mCherry-M.SssI) (Fig. 4a) and western blotting (C33A MLS-M.SssI, -M.CviPI and HCT116 MLS-M.SssI, -M.SssI ∆∆, -M.CviPI) (Fig. 4b). Moreover, no increase in methylation was observed in a hypomethylated nDNA region (*GAPDH*) (Suppl. Fig. 1). Important to mention is that the mitochondrial expression of M.SssI or M.CviPI was not associated with any toxicity, which is in contrast to the nuclear expression of e.g. M.SssI^33^ in mammalian cells.

### Effect of mtDNA methylation on mtDNA gene expression and copy number

**Figure 5:**
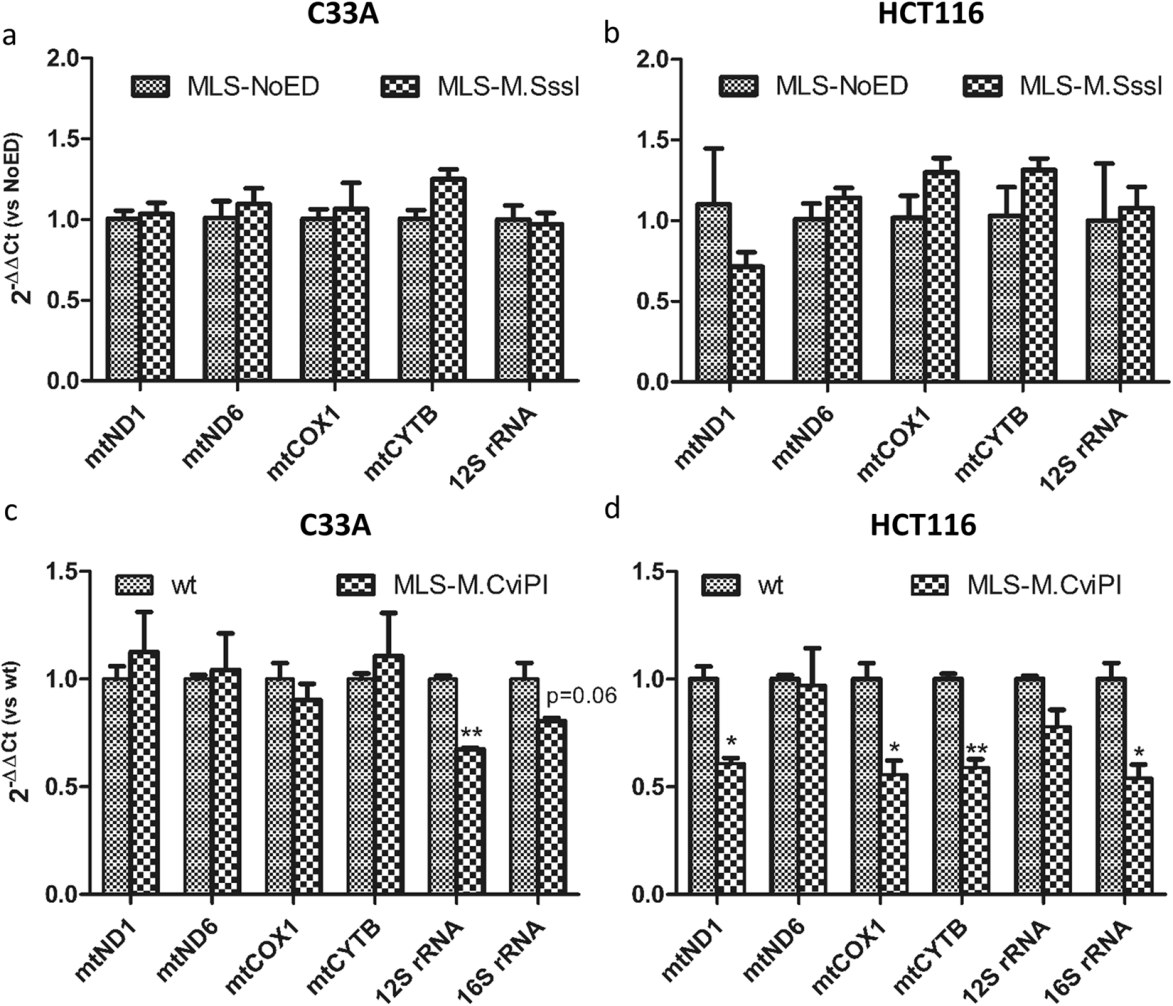
Normalised mitochondrial gene expression in cells expressing mitochondria-targeted M.SssI or M.CviPI. Expression of five (**a**,**b**) or six (**c**,**d**) mitochondrial genes (*mtND1*, *mtND6*, *mtCOX1*, *mtCYTB*, *12S rRNA* and *16S rRNA*) was determined in stable cell lines of C33A (**a**) or HCT116 (**b**) cells expressing mitochondria-targeted M.SssI (MLS-M.SssI) or empty vector control (MLS-NoED), and C33A (**c**) or HCT116 (**d**) cells expressing mitochondria-targeted M.CviPI (MLS-M.CviPI) or wild-type cells (wt). Each bar shows the mean ± SEM of three independent experiments.

In the nucleus DNA methylation is often associated with gene repression. To determine whether this holds true for mtDNA methylation, a qRT-PCR was performed on five or six mitochondrial genes: *mtND1*, *mtND6*, *mtCOX1*, *mtCYTB*, *12S rRNA* and *16S rRNA*. These genes were chosen in such a way that at least one gene of each mitochondrial promoter was interrogated (Fig. 1), i.e. LSP (*mtND6*), HSP1 (*12S* and *16S rRNA*) and HSP2 (*12S* and *16S rRNA*, *mtND1*, *mtCOX1*, *mtCYTB*). Unexpectedly, M.SssI-induced CpG methylation of the mtDNA did not significantly alter the expression of any of the genes tested in either C33A (Fig. 5a) or HCT116 cells (Fig. 5b). In contrast, M.CviPI-induced GpC methylation of the mtDNA did significantly repress a number of mitochondrial genes. Interestingly, dependent on the cell line, either the HSP1-regulated genes (C33A, Fig. 5c) or the HSP2-regulated genes (HCT116, Fig. 5d) were repressed. The effect of M.CviPI was not the result of overexpression of a mitochondrial targeting construct as no effect on gene expression was observed after targeting the empty vector to the mitochondria (MLS-NoED) (Suppl. Fig. 2).

**Figure 6:**
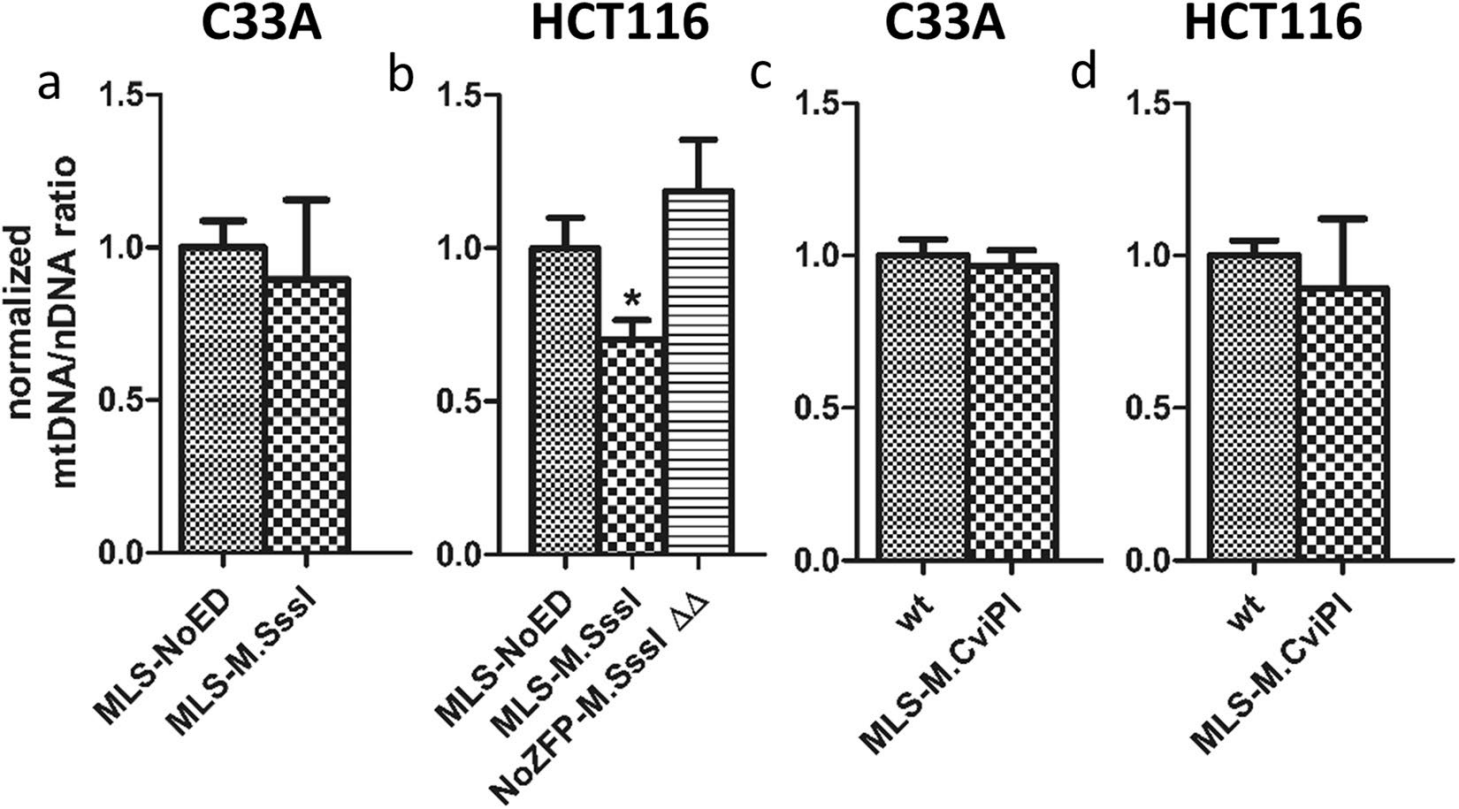
Normalised mitochondrial DNA copy number of cells expressing mitochondria-targeted M.SssI, the catalytically inactive double mutant of M.SssI, or M.CviPI. The effect of mitochondria-targeted M.SssI (MLS-M.SssI) or the catalytically inactive double mutant of M.SssI (MLS-M.SssI ∆∆, only for HCT116 cells) on mitochondrial DNA copy number normalised to empty vector control (MLS-NoED) was determined in stable cell lines of C33A (**a**) or HCT116 (**b**). Similarly, the effect of mitochondria-targeted M.CviPI (MLS-M.CvPI) on mitochondrial DNA copy number normalised to wild-type control (wt) was determined in stable cell lines of C33A (**c**) or HCT116 (**d**). Each data point represents the mean ± SEM of at least three independent experiments.

As mitochondrial gene repression can be the effect of a lower number of mtDNA molecules^23^ (Suppl. Fig. 3), we determined whether the effects on gene expression were the result of changes in mtDNA copy number (Fig. 6). MtDNA copy number was unchanged in the C33A cells expressing MLS-M.SssI (Fig. 6a) or MLS-M.CviPI (Fig. 6c), as well as in the HCT116 cells expressing MLS-M.CviPI (Fig. 6d). Therefore, the effect on gene expression induced by M.CviPI (Fig. 5c,d) seems to be the direct result of mtDNA methylation. The only condition that did result in a reduction of mtDNA copy number was in the HCT116 cells expressing MLS-M.SssI. In this condition, the relative copy number decreased to 0.70 ± 0.06 (p < 0.05) (Fig. 6b). These results were confirmed using an independent mitochondrial primer pair amplifying the *mtCOX1* region (Suppl. Fig. 3). The effect on mtDNA copy number was dependent on the DNA methyltransferase activity of M.SssI, as the catalytically inactive double mutant of M.SssI did not affect the mtDNA copy number (Fig. 6b).

**Figure 7:**
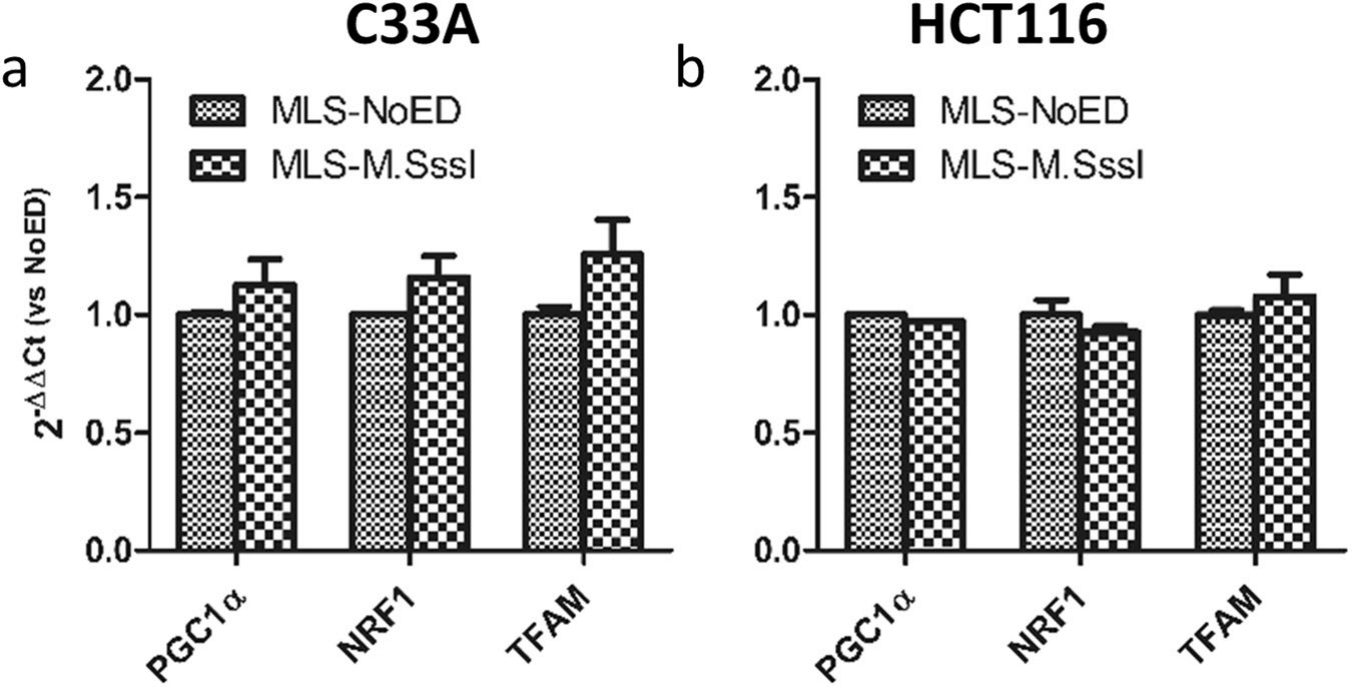
Expression of nuclear-encoded mitochondrial genes involved in mitochondrial biogenesis in cells expressing mitochondria-targeted M.SssI. Expression of four mitochondrial genes (*mtND1*, *mtND6*, *mtCOX1* and *mtCYTB*) was determined in stable cell lines of C33A (**a**) or HCT116 (**b**) cells expressing mitochondria-targeted M.SssI (MLS-M.SssI) or empty vector control (MLS-NoED). Each bar shows the mean ± SEM of three independent experiments.

To gain insight into the mechanism by which mtDNA methylation may affect mtDNA copy number, we determined the expression of three nuclear-encoded genes (*PGC1α*, *NRF1*, *TFAM*) involved in the mtDNA biogenesis (Fig. 7). In neither the C33A (Fig. 7a) nor the HCT116 (Fig. 7b) cells expressing MLS-M.SssI, gene expression of these genes was changed. Therefore, mtDNA methylation is not indirectly regulating the mtDNA copy number via the regulation of nuclear-encoded mitochondrial biogenesis genes.

**Figure 8:**
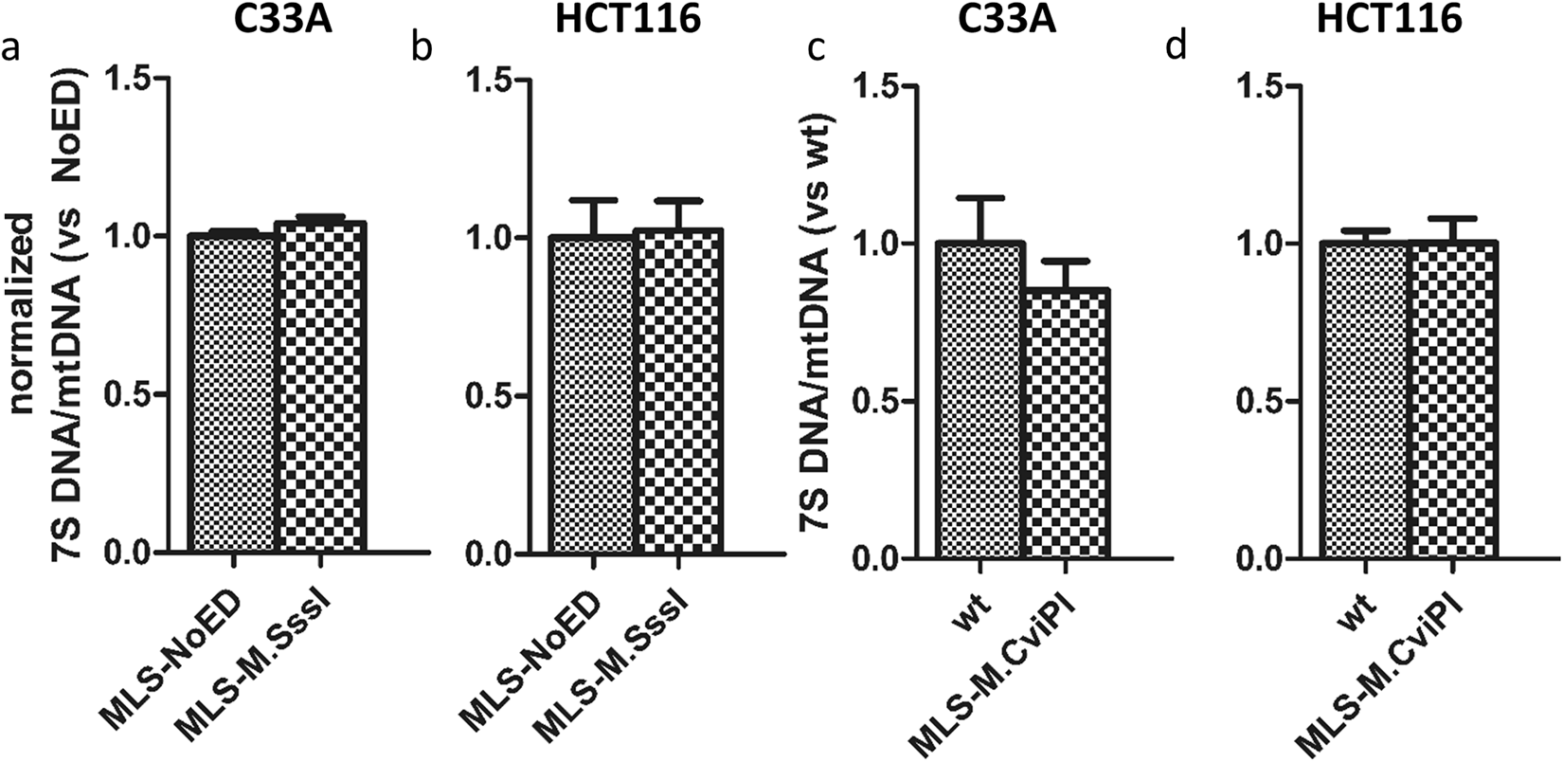
7S DNA quantification in mitochondria-targeted M.SssI or M.CviPI. The effect of mitochondria-targeted M.SssI (MLS-M.SssI) on 7S DNA primer formation normalised to empty vector control (MLS-NoED) was determined in stable cell lines of C33A (**a**) or HCT116 (**b**). Similarly, the effect of mitochondria-targeted M.CviPI (MLS-M.CvPI) on 7S DNA primer formation normalised to wild-type control (wt) was determined in stable cell lines of C33A (**c**) or HCT116 (**d**). Each data point represents the mean ± SEM of three independent experiments.

MtDNA methylation has been suggested to play a role in D-loop formation and mtDNA replication^13, 15^, possibly via the regulation of 7S DNA primer formation^15^. To address this, we studied the effect of CpG (Fig. 8a,b) or GpC (Fig. 8c,d) methylation on 7S DNA primer formation in C33A (Fig. 8a,c) and HCT116 (Fig. 8b,d) cells. However, as shown in Fig. 8, 7S DNA primer formation was not affected by CpG or GpC methylation.

**Figure 9:**
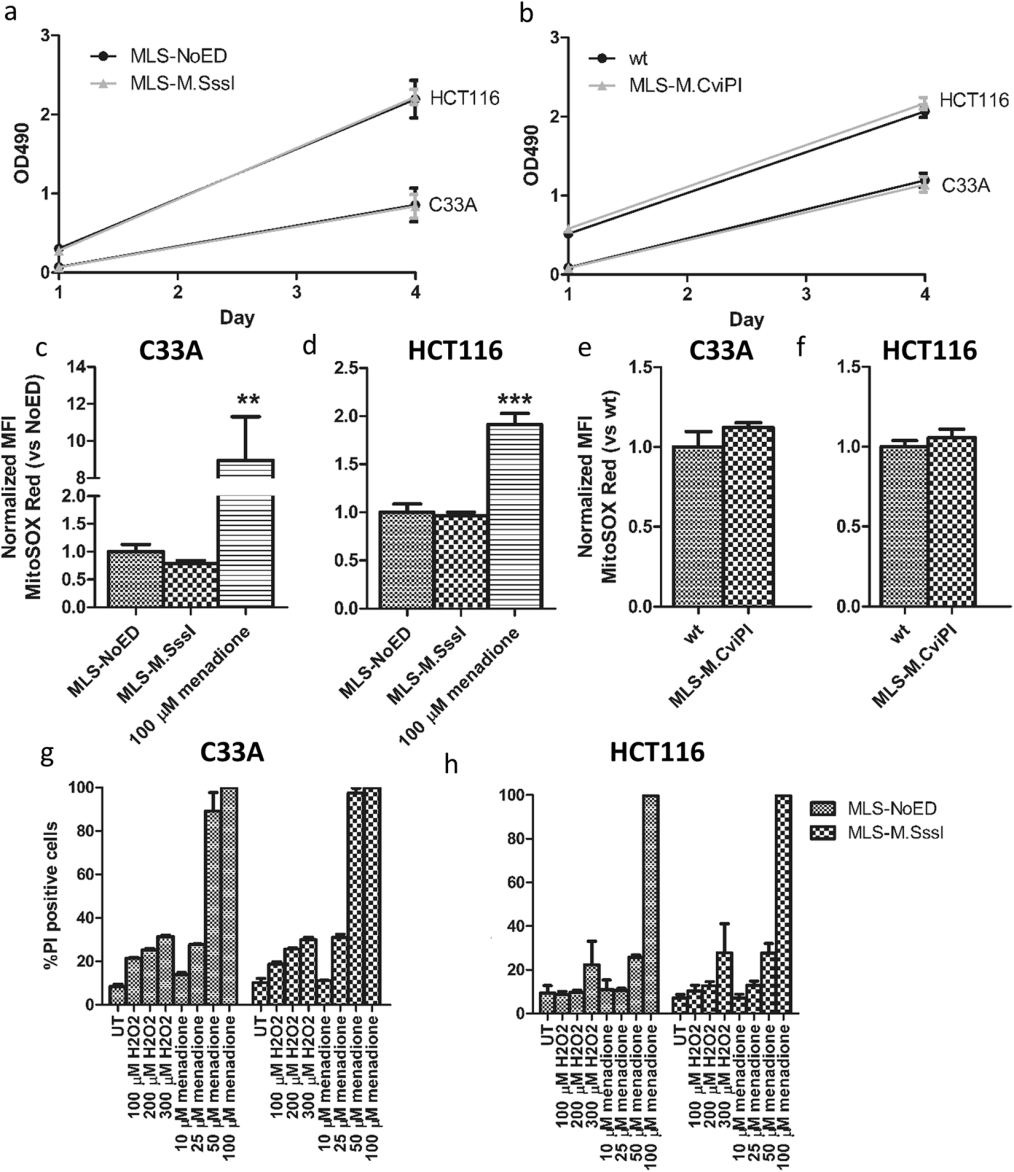
Mitochondrial and cellular functions in general in cells expressing mitochondria-targeted M.SssI or M.CviPI. In C33A (**a**,**b**,**c**,**e**,**g**) and HCT116 (**a**,**b**,**d**,**f**,**h**) cells stably expressing mitochondria-targeted M.SssI (MLS-M.SssI) or mitochondria-targeted M.CviPI (MLS-M.CviPI) the effect was determined of mtDNA methylation on: (**a**,**b**) mitochondrial metabolic activity (day 1) and cell proliferation (day 4), as measured by MTS; (**c–f**) mitochondrial superoxide (O_2_^•−^) production, as measured with the MitoSox Red ROS probe; (**g**,**h**) sensitivity toward ROS-induced cell death, as measured with PI. H_2_O_2_ was used as a general ROS inducer, whereas menadione was used to specifically induce mitochondrial O_2_^•−^. Each data point corresponds to the mean ± SEM of at least three independent experiments.

From the above, it seems that depending on the cell type (C33A vs HCT116, Suppl. Table 2) and context of cytosine methylation (CpG vs GpC), mtDNA methylation can play a role in reducing mtDNA gene expression (Fig. 5c,d) or mtDNA copy number (Fig. 6b). We wondered whether this would affect any mitochondrial or cellular functions in general. First, we tested the effect of CpG (Fig. 9a) and GpC (Fig. 9b) methylation on mitochondrial metabolic activity and cell proliferation of the stable cell lines. As becomes clear from those figures, in both cell lines, mitochondrial metabolic activity and cell proliferation were unaffected by CpG (Fig. 9a) or GpC (Fig. 9b) methylation. As mitochondrial dysfunction is often associated with a change in mitochondrial superoxide production^34^, the production of mitochondrial superoxide was assessed by the mitoSox Red ROS probe. Mitochondrial superoxide production also did not change upon induction of CpG (Fig. 9c,d) or GpC (Fig. 9e,f) methylation in C33A (Fig. 9c,e) or HCT116 cells (Fig. 9d,f). As we did not observe any downstream effects of the mtDNA methylation induced changes on mitochondrial or general functions, we hypothesized that the functional effect of mtDNA methylation may only become visible under stress conditions. Since mitochondria are major producers of reactive oxygen species (ROS)^34^, we looked into the effect of nearly complete CpG methylation on the sensitivity toward ROS-induced cell death in C33A (Fig. 9g) or HCT116 cells (Fig. 9h). Again, this function remained unchanged upon induction of CpG methylation.

## Discussion

By targeting the CpG methyltransferase M.SssI or the GpC methyltransferase M.CviPI to the mitochondria, we could show for the first time that mtDNA methylation might have a direct, be it context-dependent effect: in HCT116, but not C33A cells, induction of CpG methylation in the mtDNA resulted in a decrease in mtDNA copy number. On the other hand, induction of GpC but not CpG methylation in the mtDNA, in either C33A or HCT116 cells, resulted in repression of HSP1- or HSP2-regulated genes, respectively. Interestingly, we could not detect any change in mitochondrial (e.g. metabolic activity, mtROS production) or cellular (e.g. cell proliferation, sensitivity to apoptosis) functions in general for either type of methylation. So, the exact consequences of these effects remain to be discovered.

In the last decades, several dozens of papers have addressed the presence of mtDNA methylation^11^. In line with early reports that reported average mtDNA methylation levels of about 2–5%^3, 9^, we detected low levels of mtDNA methylation. Similarly, a recent study by Liu *et al.* found only 2 CpGs (out of the 83 analysed) to be methylated more than 5%^35^. Remarkably, these 2 CpGs were both located in the D-loop region, and the one investigated in our study, CpG 454 bp, turned out to be the most prominently methylated CpG (varying between 11–17%) of our study as well. Also two other recent studies confirmed that mtDNA methylation levels are on average very low, although regional differences across the mitochondrial genome seem to exist^8, 36^, with great inter-individual differences^36^. In contrast to all these findings, other recent papers could not confirm the presence of mtDNA methylation^6, 7^. When performing next-generation bisulfite sequencing on DNA from HCT116 cells and analysing publicly available genome-wide bisulfite sequencing data from several other DNA sources, Hong *et al.* were unable to detect mtDNA methylation^7^. Strikingly, the HCT116 cells were the only cell line for which we could not detect any methylation either. However, the obtained coverage of 94× the mtDNA may not have been sufficient to enable the detection of mtDNA methylation.

Another striking observation from literature, is the relative abundance of cytosine mtDNA methylation beyond the CpG context, i.e. CpH methylation. While in the nDNA CpG methylation is most predominant, some studies suggest that in the D-loop region of the mtDNA CpH methylation is relatively important^13, 15^. Importantly, by using the appropriate controls, such as replicates, alternative bisulfite procedures, unmethylated control samples and samples lacking mtDNA (ρ^0^ cells), the chance that this high level of CpH methylation could be contributed to incomplete bisulfite conversion or the amplification of nuclear copies of mtDNA (NUMTs), was minimised. In contrast to these two studies, we observed very low (<1% methylation) levels of CpH methylation in all cell lines tested (HCT116, C33A, SKOV3 and HeLa) and in both interrogated regions (D-loop, *mtCOX2*). Such low levels of CpH methylation cannot be distinguished from incomplete bisulfite conversion^7, 35^. In line with our observations, Blanch *et al.* also detected very low levels of CpH methylation^14^. Altogether, CpH methylation may occur in certain circumstances, but if so, the level and pattern seem to vary by unknown factors.

Despite a very low level of mtDNA methylation, various papers report on a link between mtDNA methylation and disease^11^. These studies eluted two possible functions for mtDNA methylation: the regulation of gene expression^12, 16, 18, 19^ and mtDNA replication^13, 15^. For example, Shock *et al.* showed that upregulation of mtDNMT1 (by p53 knockdown) resulted in increased expression of *mtND1*, decreased expression of *mtND6* and unchanged expression of *mtATP6* and *mtCOX1* ^12^. However, as p53 knockdown has multiple downstream effects, e.g. upregulation of nuclear DNMT1, it is impossible to exclude the possibility that these findings were due to indirect effects of the p53 knockdown or due to the increase in nuclear DNMT1 levels. Similarly, in human retinal endothelial cells from deceased patients with diabetic retinopathy, the link found between mtDNA methylation and repression of certain mtDNA genes^18^ may well be explained by other factors, including the upregulation of nuclear DNMT1 levels. As a consequence, the nuclear DNA of genes involved in mitochondrial biogenesis could have been methylated, which would explain the reduction in gene expression. Indeed, it has been described in diabetic retinopathy that the mtDNA polymerase POLG becomes hypermethylated and compromises mitochondrial transcription^37^. In contrast to such previously published approaches, our mitochondria-specific methylation-induction experiments enabled us to determine any direct downstream consequences of mtDNA methylation.

Induction of high levels of CpG methylation by M.SssI decreased the mtDNA copy number in HCT116 cells, but not in C33A cells. However, we could not clarify any cellular consequences of this reduction in mtDNA copy number for HCT116 cells. In our case, the reduction in mtDNA copy number did not result in a reduction in gene expression, as has been proposed to be one of the mechanisms regulating mitochondrial gene expression^23, 24^. Since the study was performed in stable cell lines, mtDNA methylation levels were continuously high. As such, the observed reduction in mtDNA copy number may have been either the result of, or an adaptation to survive, the high level of methylation. However, if this would have been an adaptive mechanism, we excluded the possibility that this was transcriptionally regulated by the master regulator (PGC1α) or other important players (NRF1, TFAM) of mitochondrial biogenesis. Alternatively, it was shown that the mtDNA copy number can modulate the methylation level of certain nuclear genes^38^. Interestingly, some of these genes, such as BACH2 and PRKC1B, are involved in the regulation of apoptosis in response to oxidative stress. As such, by altering the mtDNA copy number, mtDNA methylation may be a way for the mitochondria to communicate to the nucleus in case of e.g. environmental stress^39, 40^. However, in our study we could not find evidence for such a mechanism; the reduced mtDNA copy number that was induced by CpG mtDNA methylation did not result in a different sensitivity towards ROS-induced cell death.

According to several recent publications, in addition to CpG methylation, CpH methylation may be present in the mtDNA^13, 15^. As such, the induction of GpC methylation, as done by us here, may provide relevant insights into a functional role for human mtDNA methylation. In our study, induction of intermediate levels of GpC methylation by M.CviPI did not affect mtDNA copy number, but decreased the expression of certain mitochondrial genes. Remarkably, both cell lines repressed genes regulated by a different mitochondrial promoter. This may point to cell-type specific factors that can affect the outcome of mtDNA methylation. For example, HCT116 cells are p53 wild-type, whereas C33A cells are p53 mutant. Since p53 is known to repress mtDNMT1^12^, this may, via unknown mechanisms, have contributed to the final outcome of mtDNA methylation. Moreover, the mtDNA is more actively transcribed (i.e. higher level of mtDNA gene expression per mtDNA molecule) and contains a higher level of TFAM in HCT116 cells compared to C33A cells (Suppl. Table 2). As a result, mtDNA methylation may have a different outcome depending on these parameters. Although our observations thus point towards relevance of GpC methylation, future studies should determine if and how these factors may contribute to the functional outcome of mtDNA methylation.

In summary, in this paper we could show that mtDNA CpG methylation, whether it exists or not, did not influence gene expression in a similar way as it does for nDNA. If anything, CpH methylation by itself or in combination with CpG methylation, might be of direct functional relevance. If this is indeed the case, it may have great consequences given the important role of the mitochondria in health and disease^41^ and the observed differential mtDNA methylation profiles in various diseases^16–19^. However, we do have to remark that the current study used an approach that induced mtDNA methylation levels that are far above what has been detected in the endogenous situation (2–5% on average)^3, 9^. As such, the actual physiological relevance of our findings remains to be further studied. Nevertheless, this is the first study that goes beyond the mere description of just another association between mtDNA methylation and a specific clinical condition. This study will thus be the start of further investigations that address the cause-consequence effects of mtDNA methylation, which are one of the necessary next steps to progress our insights into a role of mtDNA methylation. Besides mtDNA methylation, also mtDNA hydroxymethylation and post-translational modifications of the mitochondria-localised, histone-like protein TFAM have been described^10^. Such epigenetic-like modifications are reversible and reprogrammable^42^, and hence, this could provide us with new therapeutic targets for many of the aforementioned diseases. Therefore, it is essential that future efforts should give us a greater insight in these previously unappreciated epigenetic modifications.

## Material and Methods

### Cell culture

C33A (human cervical cancer), HCT116 (human colon cancer) and HEK293T (human embryonic kidney) cells were obtained from the ATCC. OSE-C2 (immortalised human ovarian epithelial cells^43^), CiGenCs (conditionally immortalised human glomerular endothelial cells^44^), IHH (immortalised human hepatocytes^45^) and BEAS-2B ρ^0^ cells (human bronchial epithelium lacking mtDNA) were kindly provided by Dr. Richard Edmondson, Dr. Simon Satchell, Dr. Han Moshage and Dr. Roland Hoffmann, respectively.

CiGenCs and IHH were cultured onto gelatin-coated flasks, whereas BEAS-2B ρ^0^ cells were cultured onto collagen-coated flasks. All cell lines, except IHH and BEAS-2B ρ^0^ cells, were cultured in high glucose (25 mM glucose) DMEM medium (Lonza) supplemented with 10% FCS (Perbio Hyclone), 2 mM L-glutamine (BioWhittaker) and 50 μg/mL gentamicin sulfate (Invitrogen). Additionally, the IHH medium contained 20 mU/ml insulin (Novo Nordisk) and 50 nmol/L dexamethasone (Sigma). The BEAS-2B ρ^0^ cells were cultured in high glucose DMEM medium supplemented with 25% FCS, 2 mM L-glutamine, 1% P/S, 2.5 μg/ml amphotericin B (Sigma), 1× MEM amino acids solution (Sigma), 1× MEM non-essential amino acid solution (Sigma), vitamins (Sigma), 50 μg/ml uridine (Sigma). All cells were kept at a humidified incubator with 5% CO_2_ at 37 °C.

For high versus low glucose treatment, cells were washed twice with PBS and were cultured for 4 days on either low (5 mM) or high (25 mM) glucose DMEM.

### Cloning

The mitochondria-targeted proteins were all cloned using one “master synthetic construct”. This “master synthetic construct” was synthesised at Bio Basic Canada and contains (from 5′- to 3′-end): 1. Kozak sequence; 2. N-terminal 49-aa mitochondrial localization signal (MLS) of the F1β subunit of mitochondrial ATP synthase^46^; 3. open position 1; 4. HA-tag; 5. 17-aa flexible linker – (SGGGG)_3_SS^46^; 6. open position 2 for epigenetic enzyme; 7. C-terminal 18-aa nuclear export signal (NES) of the nonstructural protein 2 of minute virus of mice^47^; 8. stopcodon. The addition of restriction enzymes between the individual components enabled flexibility in cloning of the mitochondria-targeted proteins: BamHI – Kozak – MLS – NruI…AvrII – Open position 1 – BsIWI…NruI – HAtag – flexible linker – EcoRV…AscI – Open position 2 – PacI…EcoRV – NES – stopcodon – NotI. This master construct was subcloned into pCDH-CMV-MCS-EF1-copGFP (CD511B-1) using BamHI and NotI restriction sites (System Biosciences). In this plasmid EF1-copGFP was swopped with SV40-puromycin resistance using NotI and XhoI restriction enzymes. An additional NES was cloned into the final construct. Moreover, the “open position 1” was removed using NruI digestion. For visualization of the plasmid, the same restriction enzymes were used to subclone mCherry into “open position 1”. All constructs as described below were cloned into this plasmid. As a negative control, a no effector domain (NoED) construct containing no protein in the “open position 2”, was generated using EcoRV digestion.

### DNMT1

To obtain a PCR product of the mitochondria-targeted DNMT1 transcript variant (mtDNMT1) from human reference cDNA (Clontech, random-primed) or a random-primed cDNA pool of human cell lines (HEK293T, HCT116, HeLa, IHH, SiHa, Caski, SKOV3, HepG2, C33A, OSE-C2), primers (BclI-mtDNMT1-NotI) as described in Table 1 were used. The amplification of mtDNMT1 was unsuccessful, despite the use of a wide range of strategies: different DNA polymerases were used according to the manufacturer’s protocol (Phusion high-fidelity DNA polymerase (Thermo Scientific), Pfu DNA polymerase (Thermo Scientific), Taq DNA polymerase (Thermo Scientific)), the composition of the PCR-mix was varied (buffer type, concentration of MgCl_2_, addition of DMSO), different PCR protocols (melting temperatures, elongation times, number of cycles, etc.) were tested. Therefore, as an alternative, the coding sequence of the normal (non-mitochondria-targeted) *DNMT1* gene (cDNA clone MGC:161505 IMAGE:8991943) was obtained using primers (AscI-DNMT1-PacI) as described in Table 1. In order to achieve mitochondria-targeting of DNMT1, this PCR product was cloned into pCDH-CMV-master synthetic construct-SV40-puro using AscI and PacI restriction sites, resulting in MLS1x-HAtag-flexible linker-DNMT1-2xNES.

### M.SssI, M.CviPI, hM.CviPII and the *E*. *coli* conII promoter

The plasmid containing M.SssI^48^ and its catalytically inactive double mutant (E186A, R230A), M.SssI∆∆^30^, were previously obtained from Dr. Antal Kiss. Plasmids containing M.CviPI^31^ and hM.CviPII^32^ were kindly provided by Dr. Michael Kladde. Before the non-human DNA methyltransferases were cloned into pCDH-CMV-master synthetic construct-SV40-puro, the *E*. *coli* conII promoter was included in the reverse orientation immediately behind the NES. This was done by annealing of a complementary pair of oligonucleotides containing conII and digested NotI fragments (Table 1). In short, equimolar concentrations of the forward and reverse oligonucleotides were mixed with NEB Buffer 4 and incubated in a waterbath at 95 °C for 5 min. By turning off the waterbath, the oligonucleotides were allowed to slowly cool down to RT. Annealed oligonucleotides were used in subsequent cloning procedures. The convergent transcription of conII relative to the DNA methyltransferase gene reduces toxicity due to leaky expression even in *E*. *coli* strains lacking methylation-dependent restriction^49^. Subcloning of M.SssI using AscI and PacI restriction enzymes, or the PCR product of M.CviPI and hM.CviPII containing AscI and PacI restriction sites enabled the generation of the pCDH-CMV-master synthetic construct-conII-SV40-puro containing MLS1x-HAtag-flexible linker-(M.SssI/M.CviPI/hM.CviPII)-2x NES. All constructs were confirmed by colony PCR and sequencing (Baseclear). Transformation of plasmids containing non-human DNMTs was performed in *E*. *coli* ER1821 cells, all others were performed in *E*. *coli* Top10 cells.

### Viral delivery of mitochondria-targeting constructs

Lentiviral particles containing the mitochondria-targeting constructs were produced as previously described^50^. In short, HEK293T packaging cells were co-transfected using the calcium phosphate method with plasmids containing the ATF, and viral packaging plasmids containing gag/pol and the vesicular stomatitis virus G protein in a 3:2:1 ratio. Viral supernatant was collected 48 h and 72 h post transfection and was used in combination with 6 µg/ml polybrene (Sigma-Aldrich) to infect C33A and HCT116 host cells. Three days after transduction, stable cell lines were generated using 1 μg/ml puromycin (Sigma) selection for 5 days. Selection medium was refreshed every 2 days.

### Validation primers

All primers used to amplify mtDNA were confirmed on agarose gel to specifically amplify the mtDNA, and not so-called NUMTs, nuclear copies of mtDNA. For this, the DNA of BEAS-2B ρ^0^ cells, containing no mtDNA, was used as negative control. For each q(RT)-PCR primer pair a standard curve was generated to calculate the efficiency of the primer pair (Suppl. Fig. 4).

### Quantitative real-time PCR (qRT-PCR)

Total RNA was isolated using the GeneJET RNA purification kit (Thermo Scientific) following manufacturer’s protocol, including an additional 15 minute DNaseI (Roche) treatment to remove DNA contamination. RNA was quantified using a Nanodrop 1000 spectrophotometer (Thermo Scientific). 1 µg of RNA was reverse transcribed into cDNA using random hexamer primers with the QuantiTect Reverse Transcription Kit (Qiagen), according to manufacturer’s protocol. Each qRT-PCR reaction contained 500 nM of each primer pair, 10 ng of cDNA and 1xABsolute qPCR SYBR Green, Rox Mix (Thermo Scientific). Primers were newly designed, extracted from the Real Time PCR primer Data Bank (RTPrimerDB, http://medgen.urgent.be/rtprimerdb/) or obtained from literature^23, 51, 52^ (Table 1). qRT-PCR reactions were conducted on the ViiA7 Real time PCR (Applied Biosystems) for 15 min at 95 °C, followed by 40 cycles of 15 sec at 95 °C, 30 sec at 60 °C and 30 sec at 72 °C. β-actin was used as housekeeping gene. Data and melting curves were analysed using ViiA7 RUO software and relative expression compared to controls was calculated using the ∆∆Ct method^53^.

### DNA isolation

Cell lysis was performed O/N at 55 °C in TNE lysis buffer (10 mM Tris/HCl, pH 7.5; 150 mM NaCl; 10 mM EDTA; 1% SDS) and 100 µg proteinase K. The following day DNA was isolated as described previously^50^. In short, lysed cells were mixed for 15 sec. with saturated (6 M) NaCl in a 5:1 ratio. This mixture was combined with an equal volume of chloroform/isoamyl alcohol (24: 1) and mixed for 60 min. on a rotor, followed by centrifugation for 20 min at 10,000 rpm at 4 °C. Total cellular DNA (genomic and mitochondrial DNA) was extracted using chloroform/isoamyl alcohol (24: 1), RNAse A (Thermo Scientific) treated for 1 h at 37 °C, and precipitated using isopropanol. DNA was quantified using a Nanodrop 1000 spectrophotometer (Thermo Scientific).

### Mitochondrial DNA (mtDNA) copy number and 7S DNA primer formation

10 ng of total cellular DNA was used as input for the qPCR. Primers amplifying a nDNA region (β-actin) and a mtDNA region (D-loop) were used (Table 1). For validation, an independent mtDNA primer pair of the mtCOX1 region was used. qPCR reactions were conducted on the ViiA7 Real time PCR (Applied Biosystems) for 15 min at 95 °C, followed by 40 cycles of 15 sec at 95 °C, 30 sec at 60 °C and 30 sec at 72 °C. Data and melting curves were analysed using ViiA7 RUO software. The mtDNA copy number was determined with the formula: $2({}^{{\rm{Ct}}}{\rm{n}}{{\rm{DNA}}}^{\ast {\rm{primer}}{\rm{efficiency}}}-{}^{{\rm{Ct}}}{\rm{m}}{{\rm{tDNA}}}^{\ast {\rm{primer}}{\rm{efficiency}}})$ ^54^. To determine the 7S DNA primer formation, primers amplifying both the mtDNA and 7S DNA (7S DNA A + B1), or only the mtDNA (7S DNA A + B2) were used (Table 1), as described previously^15^. The level of 7S DNA was calculated with the formula: $2({}^{{\rm{Ct}}}7{\rm{S}}\,{\rm{DNA}}\,{\rm{A}}+{\rm{B}}{2}^{\ast {\rm{primer}}{\rm{efficiency}}}-{}^{{\rm{Ct}}}7{\rm{S}}\,{\rm{DNA}}\,{\rm{A}}+{\rm{B}}{1}^{\ast {\rm{primer}}{\rm{efficiency}}})$.

### Bisulfite sequencing

400 ng DNA was bisulfite converted using the EZ DNA methylation Gold kit (Zymo Research) according to manufacturer’s instructions. Bisulfite PCR of the D-loop^13^ and mtCOX2^7^ was performed using bisulfite-specific primers (Table 1) as described previously^7, 13^. PCR products were cloned into pCR4-TOPO vector (Thermo Scientific) and individual clones were send for sequencing. Bisulfite sequencing results were analysed using the online tool QUMA (www.quma.cdb.riken.jp/)^55^.

### Methylated DNA immunoprecipitation (MeDIP)

For each immunoprecipitation, 1 µg of total cellular DNA was sonicated using the Bioruptor Pico (20 cycles of 20″ on, 40″ off). 5 mC DNA immunoprecipitation was performed using the Methylamp methylated DNA capture kit (Epigentek) according to manufacturer’s instructions. DNA immunoprecipitation using a normal mouse IgG antibody was performed as negative control. The enrichment of 5 mC in specific mtDNA regions was analysed using primers for the *D-loop*, *mtCYTB*, *mtCOX2* (as described before in ref. 18, Table 1).

### Confocal microscopy

Localization of the mCherry-mitochondria-targeting M.SssI fusion construct was visualised using confocal fluorescent microscopy (Leica SP8, HC PL APO CS2 63×/1.4 lens). Following manufacturer’s recommendations, to stain the mitochondria, cells were treated with 100 nM Mitotracker Deep Red FM (Molecular Probes) for 30 min at 37 °C. The mCherry-mitochondria-targeting M.SssI fusion protein was excited using a 552 nm laser light and Mitotracker Deep Red was excited using a 633 nm laser light.

### Western blotting

Cells were collected in resuspension buffer (100 mM NaCl, 15 mM MgCl_2_, 100 mM Tris, pH 7.5) and incubated on ice for 10 min while vortexing regularly. Samples were homogenised by flushing the cells 5 times through a G25 needle. Subsequently, nuclear (NER) and mitochondrial (MER) protein fractions were collected using differential centrifugation^56^. Protein quantification was performed with the DC BioRad Protein Assay (BioRad). 50 µg protein was loaded on a 12% SDS-PAGE gel for the detection of the mitochondria-targeting construct (containing a HAtag). Blots were blocked for 1 h with 5% skimmed milk in TBS. For detection, primary antibodies were incubated O/N at 4 °C, whereas secondary antibodies were incubated for 1 h at RT. The following antibodies were used: 1:1000 mouse anti-HAtag (HA.11, Biolegend), 1:1000 rabbit anti-VDAC1/Porin (Ab34726, Abcam), 1:1000 mouse anti-lamin B1 (clone L5, Invitrogen), and 1:1000 horseradish peroxidase-conjugated rabbit anti-mouse (P0260, Dako) and swine anti-rabbit (P0217, Dako). Western blot signal was generated with Pierce ECL Plus Western blot substrate (Thermo Scientific) and detected with the Biorad ChemiDoc MP imaging system (Biorad).

### Mitochondrial metabolic activity

Cells were seeded in 96-wells plates at a density of 3200 cells per well. The MTS assay was used to determine mitochondrial metabolic activity and cell proliferation^57^. In short, one day (for the mitochondrial metabolic activity) or four days (for the cell proliferation) after seeding, CellTiter 96 Aqueous One solution (Promega) was added to each well and incubated for 3 h at 37 °C. Then, the absorbance was detected at 490 nm with a Versamax microplate reader (Molecular Devices). When measuring the absorbance one day after seeding equal cell numbers, any difference in absorbance can only be explained by a difference in mitochondrial metabolic activity, whereas four days after seeding, any difference in absorbance could be the result of differences in mitochondrial metabolic activity and cell proliferation/cell viability.

### Mitochondrial ROS production

Mitochondrial superoxide levels were determined using the MitoSOX Red ROS probe. Cells were washed twice with phenol-red free DMEM, and incubated with 5 μM MitoSOX Red in phenol-red free DMEM for 30 min at 37 °C. After treatment, cells were trypsinised and collected for FACS measurements (BD LSR-II, BD Biosciences) using a 355 nm UV-laser in combination with a 575/26 nm filter^58^. As a positive control, cells were treated with 100 µM menadione for 1 h at 37 °C.

### Cell death analysis

As previously described^59^, sensitivity toward ROS-induced cell death was determined using propidium iodide (PI) as marker for late apoptotic/necrotic cells. Cells were stained for 10 min with 5 μg/mL PI (Sigma-Aldrich) in PBS at 4 °C in the dark. PI fluorescence was measured using the FL-3 channel of a FACScalibur flow cytometer (Beckton Dickenson Biosciences). The percentage of PI positive cells was determined with Kaluza 1.2 (Beckman Coulter) software and graphs were made using Graphpad Prism 5 software (GraphPad Software Inc.).

### Statistical analysis

All experiments were performed three times, unless stated otherwise. Statistical analysis was performed using Graphpad Prism 5 software. Single group and multiple group comparisons were performed with the student’s t-test or one-way ANOVA followed by Dunnett’s post hoc test, respectively. A p-value of 0.05 or less was considered statistical significant (*p ≤ 0.05, **p < 0.01 and ***p < 0.001).

## Electronic supplementary material

Supplementary figures
