# Supplementary material for: Experimental mitochondria-targeted DNA methylation identifies GpC methylation, not CpG methylation, as potential regulator of mitochondrial gene expression
Source: Sci Rep. 2017 Mar 14;7:177. doi: 10.1038/s41598-017-00263-z (PMC5428053; doi:10.1038/s41598-017-00263-z)
Supplement: Supplementary file 1 — Supplementary figures [file 41598_2017_263_MOESM1_ESM.pdf]

Supplementary information

Experimental mitochondria-targeted DNA methylation identifies GpC methylation, not CpG methylation, as potential regulator of mitochondrial gene expression.

Monique G.P. van der Wijst, Amanda Y. van Tilburg, Marcel H.J. Ruiters, Marianne G. Rots

Supplementary Figure 1 van der Wijst et al

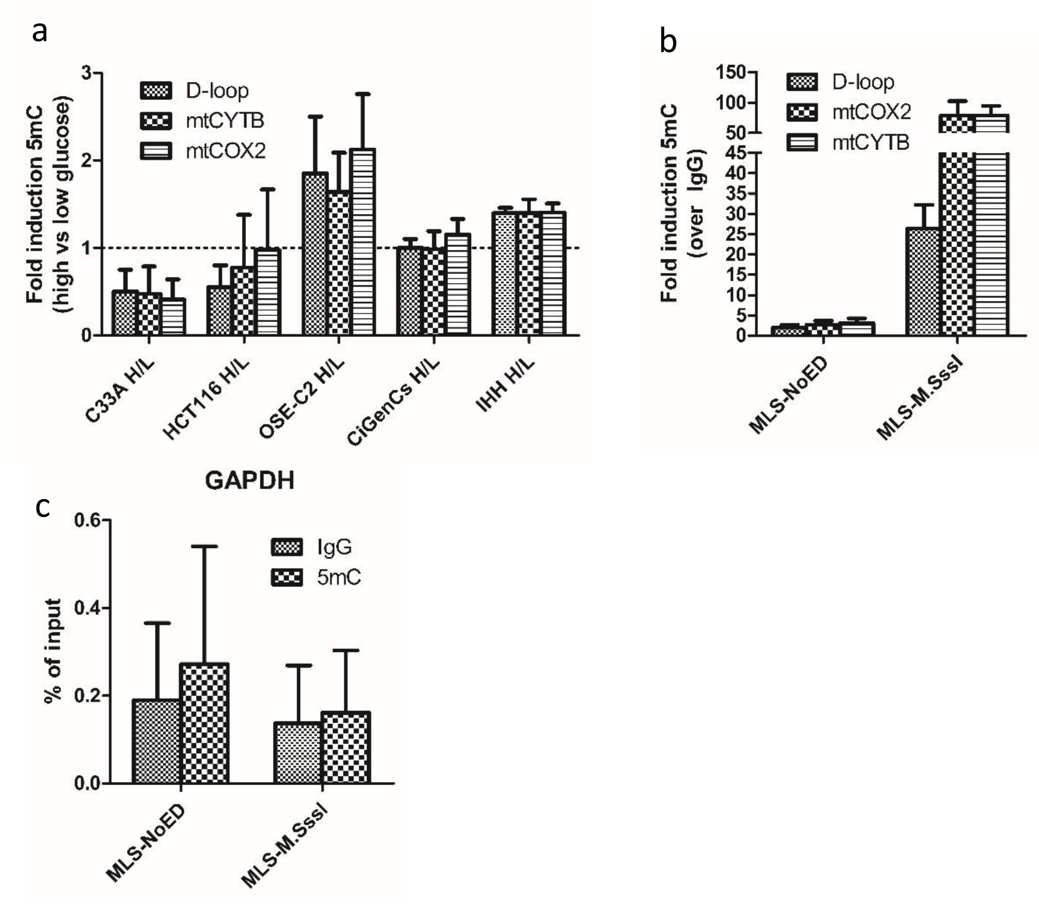

Supplementary Figure 1. Methylated DNA immunoprecipitation (MeDIP) of three mtDNA regions. (**a**) Cancerous (C33A, HCT116) and normal (OSE-C2, CiGenCs, immortalized human hepatocytes (IHH)) cell lines were exposed to high (H, 25 mM) versus low (L, 5 mM) glucose for 4 days. Thereafter, their mtDNA methylation level was determined in three mtDNA regions (D-loop, mtCYTB, mtCOX2). (**b**) The mtDNA methylation level of HCT116 cells stably expressing mitochondria-targeted M.SssI (MLS-M.SssI) or empty vector (MLS-NoED) was determined in three mtDNA regions (D-loop, mtCYTB, mtCOX2) and a (**c**) hypomethylated nuclear DNA region (GAPDH, N=2) using a MeDIP approach.

Supplementary Figure 2 van der Wijst et al

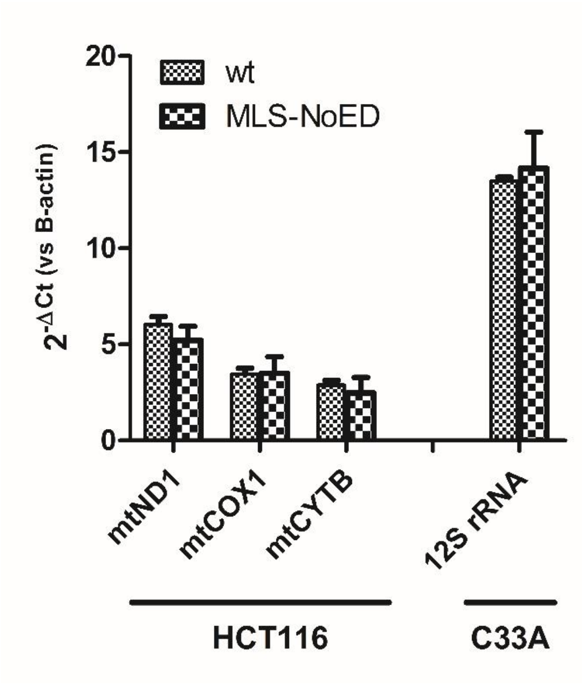

Supplementary Figure 2. Gene expression relative to β-actin. The effect of our mitochondria-targeting construct (MLS-NoED) on mitochondrial gene expression was determined compared to wild-type (wt) HCT116 and C33A cells. Note that the MLS-NoED expressing cells were processed in a different set of experiments (M.SssI experiments) compared to the wt cells (M.CviPI experiments). Only those genes showing an effect on mitochondrial gene expression in Figure 5 and those taken along in both M.SssI and M.CviPI experiments, were compared in our analysis.

Supplementary Figure 3 van der Wijst et al

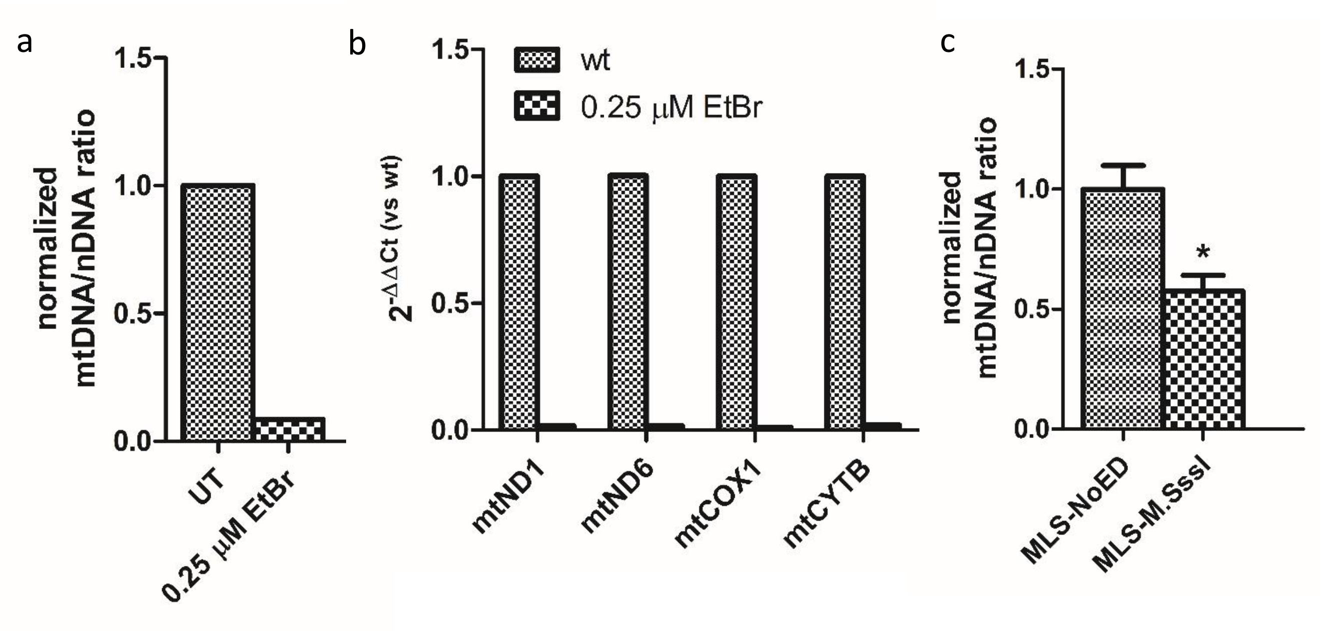

Supplementary Figure 3. Mitochondrial DNA copy number and gene expression. (**a**, **b**) As a positive control for mtDNA depletion, HeLa cells were treated for 2 days with 0.25 μM EtBr. The effect on mtDNA copy number (**a**) and mtDNA gene expression of four mitochondrial genes (*mtND1*, *mtND6*, *mtCOX1* and *mtCYTB*) **(b)** was determined (N=1). (**c**) Validation of decrease in mtDNA copy number in HCT116 cells expressing a mitochondria-targeted M.SssI (MLS-M.SssI) using an independent primer pair (mtCOX1 region).

Supplementary Figure 4 van der Wijst et al

qPCR DNA

mtDNA ratio D-loop

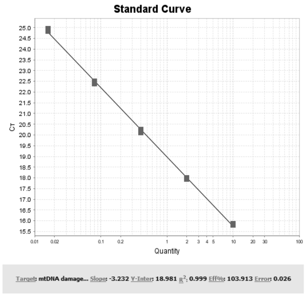

nDNA ratio β-actin

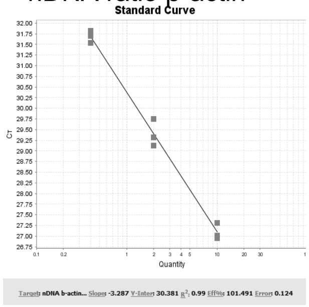

7S DNA A+B1

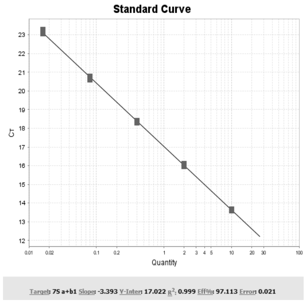

7S DNA A+B2

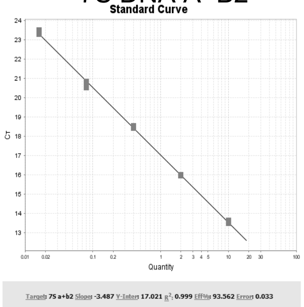

qRT-PCR mitochondrial-encoded genes

mtND1

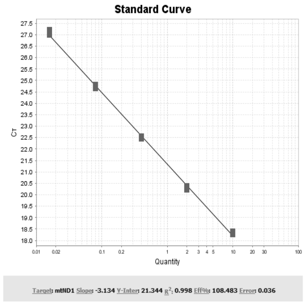

mtND6

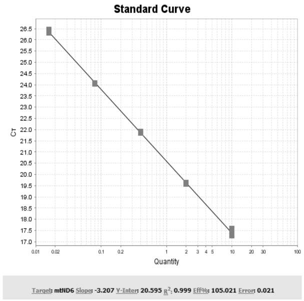

mtCOX1

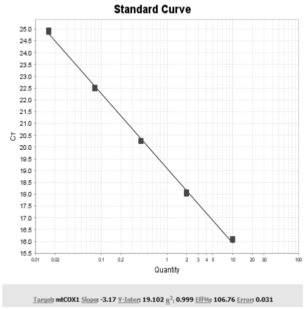

mtCYTB

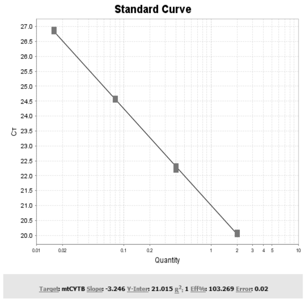

12S rRNA

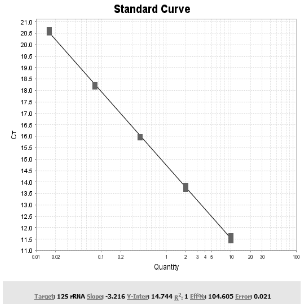

16S rRNA

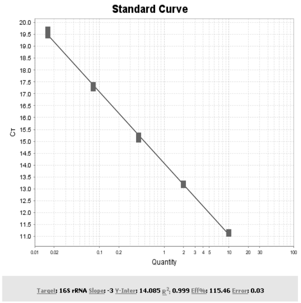

qRT-PCR nuclear-encoded genes

TFAM

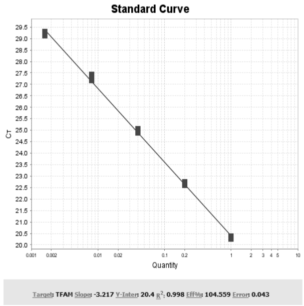

NRF1

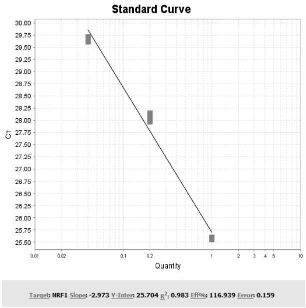

PGC1α

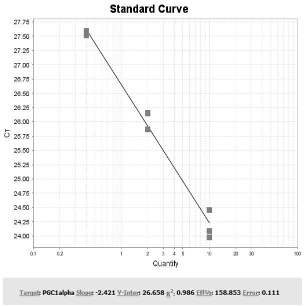

β-actin

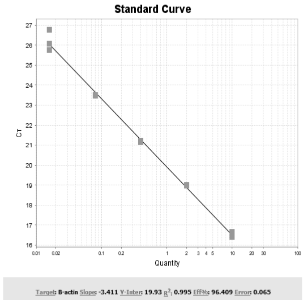

qPCR MeDIP

D-loop

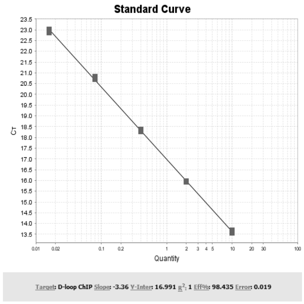

mtCOX2

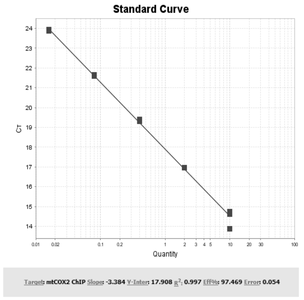

mtCYTB

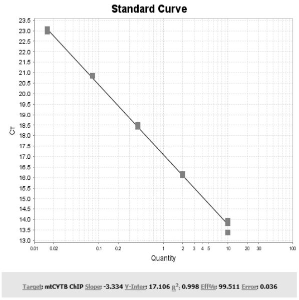

GAPDH

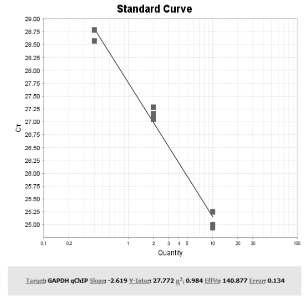

Supplementary Figure 4. Standard curves for all primers used in q(RT)-PCR.

Locus (position in mtDNA)	# of Cs	Cell line	Unconverted Cs at CpG sites	Unconverted Cs at non-CpG sites
**D-loop**<br>H: 357–621 bp	H: 99<br>(95x non-CpG,<br>4x CpG)	C33A	H: 6/109 (5.5%)<br>H: 0/108 (0%)<br>H: 6/86 (8.8%)<br>L: 0/76 (0%)	H: 8/1615 (0.5%)<br>H: 0/2565 (0%)<br><br>L: 6/1598 (0.4%)
L: 357–608 bp	L: 98<br>(94x non-CpG,<br>4x CpG)	HCT116	H: 1/40 (2.5%)	H: 1/950 (0.1%)
		HeLa	H: 0/28 (0%)<br>L: 0/24 (0%)	H: 3/376 (0.8%)<br>L: 2/564 (0.4%)
		SKOV3	H: 1/36 (2.8%)	H: 1/846 (0.1%)
**mtCOX2**<br>L: 7909–8165 bp	L: 81<br>(64x non-CpG,<br>17x CpG)	C33A	1/204 (0.5%)	1/768 (0.1%)
		HCT116	0/119 (0%)<br>0/323 (0%)	2/448 (0.4%)<br>6/704 (0.9%)
		HeLa	1/85 (1.2%)	2/256 (0.8%)

Supplementary Table 1. Overview of CpG and non-CpG methylation in the mtDNA in several cancer cell lines (analyzed by bisulfite sequencing). Each row represents an independent bisulfite sequencing batch. H, heavy strand; L, light strand.

Category	Gene/Transcript	C33A ($2^{-\Delta Ct}$)	HCT116 ($2^{-\Delta Ct}$)	Difference (HCT116/C33A)
**mtDNA OXPHOS genes**	**mtND1 (H)**	4.889	6.028	1.2x
	**mtND6 (L)**	2.511	4.260	1.7x
	**mtCOX2 (H)**	3.162	3.434	1.1x
	**mtCYTB (H)**	2.144	2.878	1.3x
**mtDNA rRNA genes**	**12S rRNA**	13.486	47.229	3.5x
	**16S rRNA**	25.865	40.303	1.6x
**Nuclear-encoded mtDNA biogenesis genes**	**PGC1α**	0.0011	0.0034	3.1x
	**NRF1**	0.012	0.021	1.8x
	**TFAM**	0.040	0.064	1.6x
	**TFAM/mtDNA**	0.0000232	0.0000347	1.5x
**DNA replication**	**7S DNA primer**	0.83	0.70	0.8x
	**mtDNA copy number**	1722	1840	1.1x

Supplementary Table 2. Overview of the differences between C33A and HCT116 cells regarding mtDNA transcription and replication. $2^{-\Delta Ct}$, relative expression to β-actin (genes), to mtDNA (7S DNA), or to nDNA (mtDNA copy number).
